# Identification of Benzothiophene-Derived Inhibitors of Flaviviruses by Targeting RNA-Dependent RNA Polymerase

**DOI:** 10.3390/v17020145

**Published:** 2025-01-23

**Authors:** Leah Liu Wang, Shazeed-Ul Karim, Aidan Hand, Ryan Brunkhorst, Mackenna Petersen, Sarah Altman, Yi Liu, Luwen Zhang, Fengwei Bai, Shi-Hua Xiang

**Affiliations:** 1 Nebraska Center for Virology and School of Veterinary Medicine and Biomedical Sciences, University of Nebraska-Lincoln, Lincoln, NE 68583, USA; 2 Department of Cell and Molecular Biology, School of Biological, Environmental, and Earth Sciences, University of Southern Mississippi, Hattiesburg, MS 39406, USA; 3 Holland Computing Center, University of Nebraska-Lincoln, Lincoln, NE 68588, USA; 4 School of Biological Sciences, University of Nebraska-Lincoln, Lincoln, NE 68583, USA

**Keywords:** flavivirus, RNA-dependent RNA polymerase (RdRp), POLcon, Benzothiophene, shared drugs

## Abstract

Flaviviruses such as Dengue, West Nile, and Zika viruses are mosquito-borne RNA viruses that can cause serious diseases in humans. To develop effective drugs for treating these viruses’ infections, we create a new approach for developing common or shared drugs that may work for several different viral species of flaviviruses. It is based on the conserved RNA-dependent RNA polymerase (RdRp), which is the key enzyme for viral replication. We built up a common structure of RdRps (POLcon) from their consensus sequence. A conserved Triple-D structural motif was identified at the active site of POLcon that has been used for virtual compound screening. We have identified three inhibitors that have potent activities against Dengue, West Nile, and Zika viruses. All these three inhibitors are Benzothiophene derivatives. This is the first report of Benzothiophene-derived compounds as inhibitors for flaviviruses. Furthermore, our approach has provided a proof-of-concept that it is feasible to identify shared drugs for several different viral species of flaviviruses.

## 1. Introduction

Flavivirus (renamed Orthoflavivirus) is a genus of the family *Flaviviridae* which includes five well-known arthropod-borne viruses: Dengue virus (DENV), West Nile virus (WNV), Zika virus (ZIKV), Japanese encephalitis virus (JEV) and yellow fever virus (YFV) [1,2]. They are transmitted by mosquitos and can cause severe diseases in humans and pose a great threat to public health [3,4]. Flaviviruses contain a single positive-stranded RNA genome of 10–11 kb, which is translated to a single large polypeptide (~3400 aa) containing three structural proteins, capsid (C), membrane protein (prM), and envelope protein (E), and seven non-structural (NS) proteins, NS1, NS2A, NS2B, NS3, NS4A, NS4B, and NS5 [5,6]. Non-structural protein 5 (NS5) consists of two enzymes, an N-terminal methyltransferase (MTase) and a C-terminal RNA polymerase, which is the RNA-dependent RNA polymerase (RdRp) [6,7,8,9]. The RdRp is the key enzyme for viral genome replication, playing a critical role in the viral life cycle. Consequently, RdRp has become a principal drug target for blocking viral replication to inhibit viral infection [10,11,12,13]. RdRp is the most conserved RNA component in the viral genomes of flaviviruses since it carries the important function of viral RNA replication [11,14,15]. Based on this exceptional property, we have analyzed different RdRps from five major viral species in the genus of flaviviruses, including DENV, WNV, and ZIKV, and built up a common 3D structure of RdRps for drug screening. In this report, we have described our first test in this approach and identified three compounds, and one of them showed potent activities against the three major flavivirus species.

## 2. Materials and Methods

### 2.1. Compounds, Viruses, and Cells

All screening compounds were purchased from the ChemBridge Corporation (San Diego, CA, USA). Each compound was dissolved at a 20 mM concentration in dimethyl sulfoxide (DMSO) and was used for stock solutions. The flaviviruses used are the Zika virus strain (MR766), which was described previously [16] and the Dengue virus Serotype 2 New Guinea C strain (DENV-2, VR-1584) were purchased from the ATCC. West Nile virus (strain CT2741) was provided by Dr. John F. Anderson at the Connecticut Agricultural Experiment Station. All viral titers were determined by plaque assay using Vero cells (African green monkey kidney cells of Vero cells ATCC CCL-81 or Vero-E6 cells, ATCC CRL-1586).

### 2.2. Virus Inhibition Assay

#### 2.2.1. Determining EC_50_ by Plaque Assay (ZIKV)

All Vero-E6 cells were seeded in a 24-well plate with a density of 10^5^ cells per well and incubated for two days. Cells were infected with virus at 0.05 PFU/cell (MOI 0.05), then incubated at 37 °C with 5% CO_2_ for 1 h shaking the plate every 10 min. The viral solution was removed after that. For screening, one concentration of compounds at 6 μM were loaded to the wells of the plate. After 48–72 h, the culture media were collected for the Plague assay.

For the EC_50_ assay, Vero-E6 cells were seeded in a 24-well plate with a density of 10^5^ cells per well. Cells were infected with viruses at 0.05 PFU/cell (MOI 0.05) and incubated at 37 °C with 5% CO_2_ for 1 h. Serial 2× diluted compound solutions were added to the cells and incubated at 37 °C with 5% CO_2_ for 48 h. Cell culture media were collected and assayed for infectious viruses by plaque assay.

#### 2.2.2. Determining EC_50_ by Plaque Assay (WNV)

Vero-E6 cells were seeded in 6-well plates at a concentration of 6 × 10^5^ cells per well. The compound (OFB3) was 2-fold serially diluted (10 μM to 0.02 μM) and applied to Vero cells for 4 h. Approximately 100 PFU of WNV were added to each well and incubated for 1 h at 37 °C with 5% CO_2_. After incubation, the compound-containing medium was removed, and the cells were covered with an overlay medium containing 1% SeaPlaque Agarose (Lonza, Morristown, NJ, USA). The plates were incubated for 4 days until the plaques were observed. Plaques were stained with Neutral Red and counted. The EC_50_ value of the compound was determined by using GraphPad Prism software (version 10.0.3).

#### 2.2.3. RT-qPCR for WNV

Vero-E6 cells were seeded in 6-well plates at a concentration of 5 × 10^5^ cells per well. After overnight incubation, Vero cells were treated with 5 μM of OFB1, OFB3, OFB15, and the control (0.1% DMSO) for 2 h. Compounds and DMSO-treated cells were then infected by 1 MOI (Multiplicity of Infection) of WNV (strain CT2741) for 24 h. After 24 h, infected cells were collected, and total RNA was extracted using Trizol Reagent. cDNA was synthesized using an Iscript cDNA Synthesis Kit (Bio-Rad, Hercules, CA, USA). WNV-envelope (WNV-E) RNA copy numbers were quantified using probe-based qPCR and normalized to cellular β-actin as a housekeeping gene [17].

#### 2.2.4. Plaque Reduction Neutralization Test (PRNT) for DENV

Vero-E6 cells were seeded in 6-well plates at a density of 6 × 10^5^ cells per well and incubated overnight. Triplicate wells of cells were treated with 10 µM of each compound, OFB1, OFB3, and OFB15, while negative control wells were treated with 0.1% DMSO for 2 h. Following treatment, all cells were infected with 100 plaque-forming units (PFU) of DENV-2 and incubated for 1 h at 37 °C with 5% CO_2_. After removing the virus inoculum, the cell monolayers were overlaid with 1% SeaPlaque agarose (Lonza) and incubated for 9 days at 37 °C with 5% CO_2_ to allow plaque formation. The plaques were stained with neutral red for 3 h, counted, and the percentage reduction in plaque formation was calculated relative to the control to evaluate the antiviral activity of the test compounds.

#### 2.2.5. Focus Forming Assay (FFA) for DENV

Vero-E6 cells were plated at 6 × 10^5^ cells/well in 6-well plates and incubated overnight at 37 °C with 5% CO_2_. The cells were treated with 10 µM of OFB1, OFB3, and OFB15 and 0.1% DMSO for negative control for 2 h at 37 °C. Then, the medium was replaced with 2 mL/well of 1 × Opti MEM GlutaMAX (Gibco, Carlsbad, CA, USA) medium supplemented with 1% methylcellulose (Sigma, St. Louis, MO, USA), 10% FBS, and 1% P/S, and incubated for 5 days. After the incubation, the overlay medium was removed and washed gently with PBS. The plates were fixed with 4% PFA for 15 min, permeabilized with 0.1% Triton-X for 20 min at RT, and blocked with 5% skim milk for 1 h. The cells were then probed with 4G2 antibody diluted with 5% skim milk in a 1:50 ratio, incubated at 4 °C overnight in the dark, followed by two 5 min washes with 0.1% PBST. The cells were then probed with goat anti-mouse IgG conjugated with horseradish peroxidase (HRP) (Abcam, catalog number ab97023, Waltham, MA, USA), diluted with 5% skim milk in 1:500 ratio, incubated at 4 °C in the dark for 2 h, followed by two 5 min washes with 0.1% PBST, and air-dried for 20 min at RT. The immuno-positive foci were developed with TrueBlue peroxidase substrate (KPL, Sera Care, Gaithersburg, MD 20879, USA) 70. The foci were counted, and the percentage reduction in foci was calculated and compared to the control to evaluate the antiviral activity of the test compounds. The method is adopted from a previous study [18].

### 2.3. Cytotoxicity Assay

The cell viability was measured by an MTT assay. The Vero-E6 cells (3000/well) were seeded in a 96-well plate and incubated for 24 h at 37 °C. Media was removed and replaced with 100 μL of compound solution in triplicates for two days and then the compound solution was replaced with 100 μL complete DMEM for continued culturing for one more day. The cultured media were removed, and the cells were washed once with PBS for analysis. A 50 μL solution of 5 mg/mL MTT [3-4,5-dimethylthiazol-2-yl)-2,5-diphenyltetrazolium bromide] (Sigma-Aldrich, St. Louis, MO, USA) was added to each well. The plates were incubated for 3 h at 37 °C, and the absorbance was measured at 570 nm and at 650 nm (background) wavelength.

### 2.4. Bioinformatic Analysis and Molecular Modeling

The sequence comparison, alignment, and dendrogram were built using BioEdit (BioEdit.exe). Molecular modeling was conducted using Modeller [19] and Discovery Studio Visualizer (Dassault Systemes BIOVIA). The consensus protein sequence of POLcon was generated from all five RdRp protein sequences, and then a POLcon 3D structure was created. A conserved three aspartic acids (Triple-D) structural motif was identified for targeting. Since non-structural protein 5 (NS5) of the DENV genome consists of two functional domains, the N-terminal methyltransferase (MTase) domain (1–272 aa) and C-terminal RNA-dependent RNA polymerase (RdRp) domain (273–900 aa) [20,21]. In the POLcon model, only the RdRp protein sequence is used and numbered, so the Triple-D motif residue positions should be D261 (D535), D391 (D665), and D392 (D666).

### 2.5. Molecular Docking

Molecular docking and analysis were performed using AutoDock Vina v1.2.6 [22,23] and open-source PyMOL [24]. AutoDock Vina is an open-source tool for effective ligand-receptor docking by estimating the standard chemical potentials between receptors and ligands [22]. Open-source PyMOL is a free version of the molecular analysis software PyMOL maintained by Schrödinger, LLC (New York, NY, USA), providing tools for creating molecular graphics and analyzing molecular interactions.

Specifically, the receptor and ligands were first preprocessed using open-source PyMOL. Then, AutoDock Vina was employed to estimate the docking results. The receptor used for docking was the POLcon with preprocessing, including the addition of hydrogen and the removal of water molecules. The grid box for the active site was positioned based on the Triple-D motif locations of POLcon. The ligands used for docking were OFB1, OFB3, and OFB15 with preprocessing, including the addition of hydrogens.

### 2.6. Statistics Assay

GraphPad Prism software (version 10.0.3) was used for all statistical analyses for making the neutralization and cytotoxicity figures and determining average values, standard errors, and values of EC_50_ or CC_50_. Statistical significance analysis was performed using a parametric unpaired *t*-test.

## 3. Results

### 3.1. Building Up the Consensus RdRp Structure (POLcon) for Compound Screening

**Figure 1:**
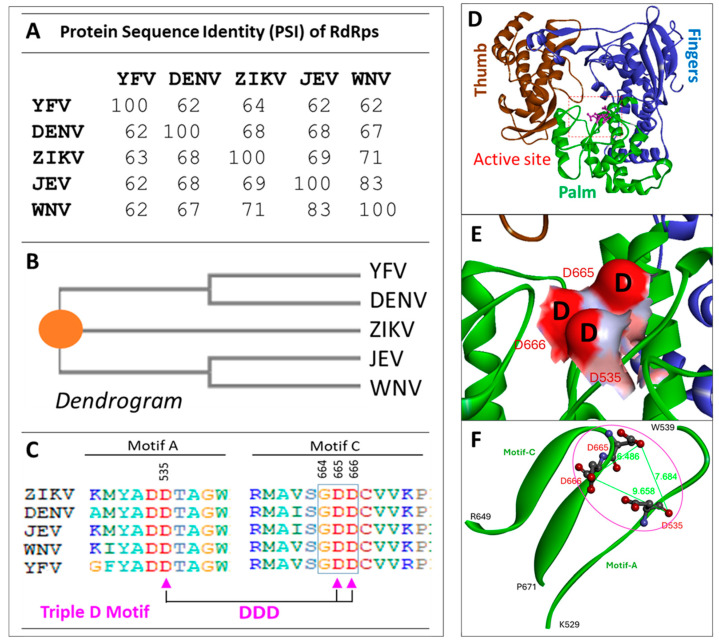
Protein sequence analysis of RdRps from flaviviruses, ZIKV (5WZ3) [25], DENV (Serotype-2, 5K5M) [26], JEV (4MTP) [27], WNV (2HFZ) [28], and YFV (6QSN) [15]. (**A**) Protein sequence identity (PSI) of five flavivirus RdRps. (**B**) Dendrograms that are generated from the five protein sequences. The genetic distances are shown in brackets. The central point is depicted as an orange circle. (**C**) The conserved Triple-D (Asp) motif sequences are in red within the sequences of five viruses. The amino acid sequence number of Triple-D (Asp) motif are based on full-length NS5 protein of DNEV-2, but the numbers based on the RdRp (POLcon) model are in the brackets: D535 (D261), D665 (D391), and D666 (D392). (**D**) The domains (Fingers, Thumb and Palm) and the Active site and Triple-D motif locations of POLcon. (**E**) Triple-D motif surface model, with negative charge surface in red. (**F**) Structural characterization of the Triple-D motif which shows location at Motif A and Motif C and the distances among these three residues are labeled.

By comparing the protein sequences of RdRps of the major human pathogenic flaviviruses, we found that their sequence identities are from 62% to 83% (Figure 1A). From the dendrogram, ZIKV is actually in the middle of two closed groups of DENV-YFV and JEV-WNV (Figure 1B). From the sequence and structural analyses of the active-site, we have identified a conserved three Aspartic acids (D535-D665-D666) motif, named Triple-D motif. Unlike the GDD motif (G664-D665-D666) described previously [8], which is a linear motif and only located the Motif C; the Triple-D motif is composed of sequences from Motif A and Motif C (Figure 1C), which is a three-dimensional structural motif and has a negatively charged surface (Figure 1E,F). We utilized their consensus sequence of the five RdRp sequences to build up a common RdRp structural model called POLcon (Figure 1D). In this model, we found the Triple-D motif appeared to be more evident in the Active site as it forms a negatively charged inner surface right at the RNA exit (Figure 1E,F), which provides a perfect drug target. Then, we evaluated this model by conducting virtual compound screening against the Triple-D motif at the active site of POLcon to identify compounds that may have activities for anti-flaviviruses.

### 3.2. Screening of Compounds Against ZIKV Infection

**Figure 2:**
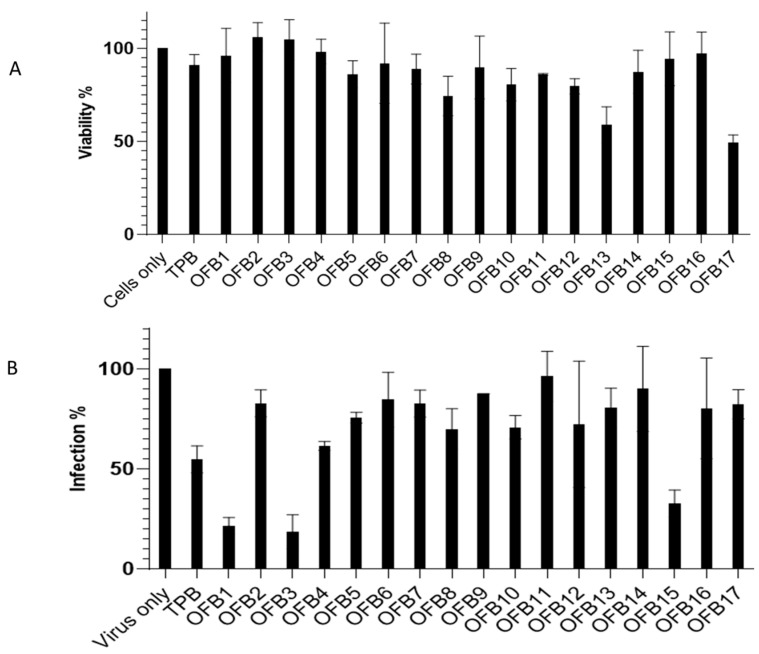
Compound screening of all 17 OFB compounds (Table S1) at 6 μM concentration against ZIKV. (**A**) Cytotoxicity assay at 6 μM using MTT method. Virus only as the positive control; TPB as a testing control. (**B**) Inhibition assay at 6 μM concentration against ZIKV; TBP as a testing positive control. Cells only as negative control.

Seventeen compounds (Table S1) from virtual screening were purchased and applied for the first experimental screening against ZIKV replication in a cell-based system. Vero-E6 cells were used for compound inhibition test using a plaque-forming assay in a 24-well plate. The cytotoxicity of the compounds was also evaluated using an MTT assay at a 6 μM concentration. The results are presented in Figure 2A for cytotoxicity assay (MTT) and Figure 2B for inhibition against ZIKV with a plaque-forming assay. It was observed that compounds OFB1, OFB3, and OFB15 showed strong inhibitions against ZIKV infection at a concentration of 6 μM without showing any cytotoxicity. They are better than compound TPB, which was previously identified as a ZIKV inhibitor that was used as the position control in our experiments.

### 3.3. Inhibition EC_50_ Analysis Against ZIKV

**Figure 3:**
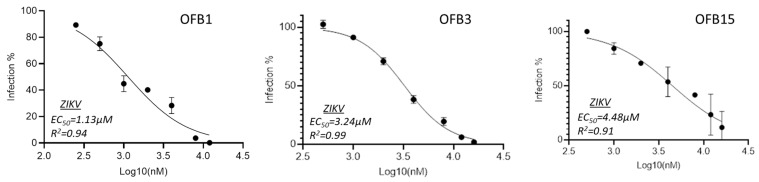
Inhibition EC_50_ assay of OFB1, OFB3, and OFB15 against ZIKV. A series of 2-fold dilutions were assessed from 0.5 μM to 16 μM, and all the tests were in triplicates.

To evaluate the inhibition potency of these three compounds, we conducted inhibition analysis using a serial concentration of compounds ranging from 0.5 μM to 16 μM to determine their EC_50_ values. The data indicated that all three compounds, OFB1, OFB3, and OFB15 showed EC_50_ values in the lower micromolar range, of 1.13 μM, 3.24 μM, and 4.48 μM, respectively (Figure 3).

### 3.4. Inhibition Analysis Against WNV

**Figure 4:**
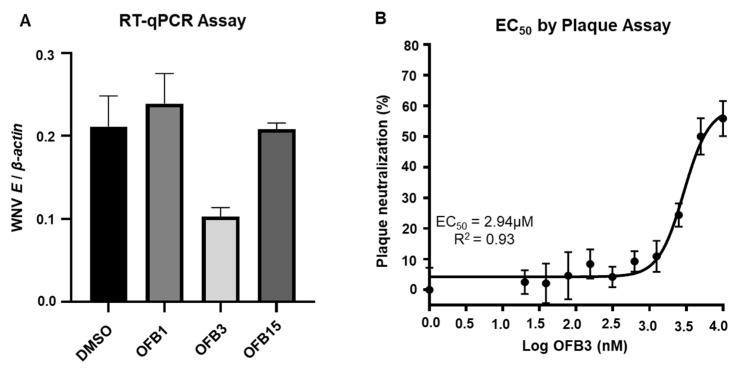
Inhibition assay against West Nile virus (WNV). (**A**) Viral load was measured using RT-qPCR from the Vero cells treated with 5 μM of OFB1, OFB3, OFB15, and the DMSO control and infected with 1 MOI WNV for 24 h. (**B**) The EC_50_ value of OFB3 through viral plaque neutralization was determined by performing plaque assay with two-fold serially diluted OFB3 compound.

To determine whether these three compounds have activity against WNV infection, we conducted an inhibition analysis. Interestingly, the screening assay at 5 μM using RT-qPCR and the results indicated that only OFB3 showed significant inhibition, but not OFB1 or OFB15 (Figure 4A). In addition, the EC_50_ analysis for compound OFB3 with plaque assay showed that the EC_50_ value is 2.94 μM (Figure 4B). It is confirmed that the compound OFB3 has a comparable inhibiting activity to WNV as to ZIKIV.

### 3.5. Inhibition Analysis Against DENV

**Figure 5:**
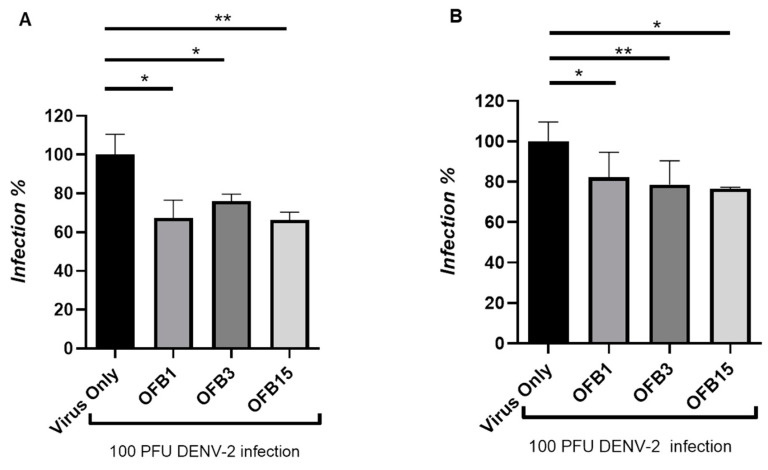
Inhibition assay of OFB1, OFB3, and OFB15 against Dengue-2. (**A**) Inhibition assay at concentration of 10 μM by Plaque forming assay (PFA). (**B**) Inhibition assay at concentration of 10 μM by Focus forming assay (FAA). * for *p* < 0.05; ** for *p* < 0.01.

DENV has four serotypes (DENV-1, DENV-2, DENV-3, and DENV-4), and all of these four serotype viruses are capable of inducing severe disease. In this report, we tested serotype 2 (DENV-2). From the plaque reduction assay, the data have indicated that all three compounds, OFB1, OFB3, and OFB15, did considerably inhibit DENV-2, but not as potently as for ZIKV at a 6 μM concentration of compounds, and the OFB3 was not as potent as for WNV, but OFB1 and OFB15 also have certain activities for WNV (Figure 5A). To confirm these data, we then used another method called focusing forming assay (FFA) which is an immunostaining-based method by detecting DENV glycoprotein with the anti-flavivirus antibody 4G2. The data are actually very similar to the plaque assay data, which demonstrated that all these three compounds (OFB1, OFB3, and OFB15) inhibit replication of the DENV-2 virus, but with less potency than against ZIKV and WNV (Figure 5B).

### 3.6. CC_50_ Assay of the Compounds OFB1, OFB3, and OFB15

**Figure 6:**
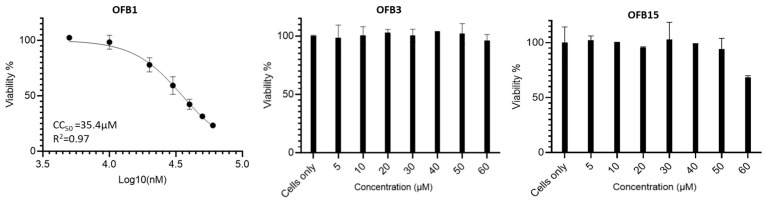
CC_50_ assay (concentration of cytotoxicity 50%) using MTT method. OFB1, OFB3 and OFB15 compounds were assessed in a serial dilution of compounds. The CC_50_ of OFB1 was determined from a serial dilution of 5, 10, 20, 30, 40, 50, to 60 µM, but OFB3 and OFB15 should be much higher than the 60 µM tested. Cells only as the positive control of cell viability.

To determine the selectivity indexes of these compounds, we conducted the cytotoxicity analysis to obtain CC_50_ values, which indicate the concentration that reduces the number of viable cells by 50% compared with the control. The CC_50_ value for OFB1 is 35.4 μM, but OFB15 started showing cytotoxicity when the concentration reached 60 μM. In contrast, OFB3 did not show any cytotoxicity when 60 μM was used, indicating that OFB3 is the safest molecule among these three compounds (Figure 6).

### 3.7. Molecular Docking of OFB1, OFB3, and OFB15

**Figure 7:**
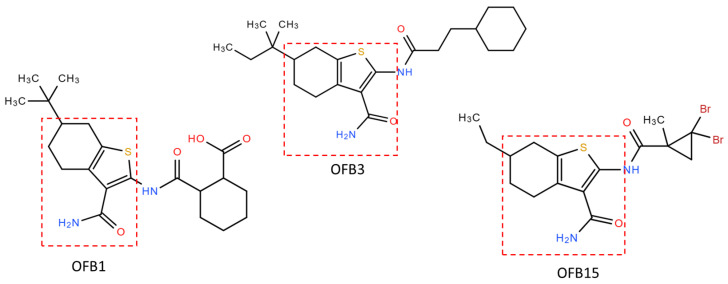
Comparisons of the compound structures of OFB1, OFB3, and OFB15. The common Benzothiophene group structure is indicated by a red dashed line.

**Figure 8:**
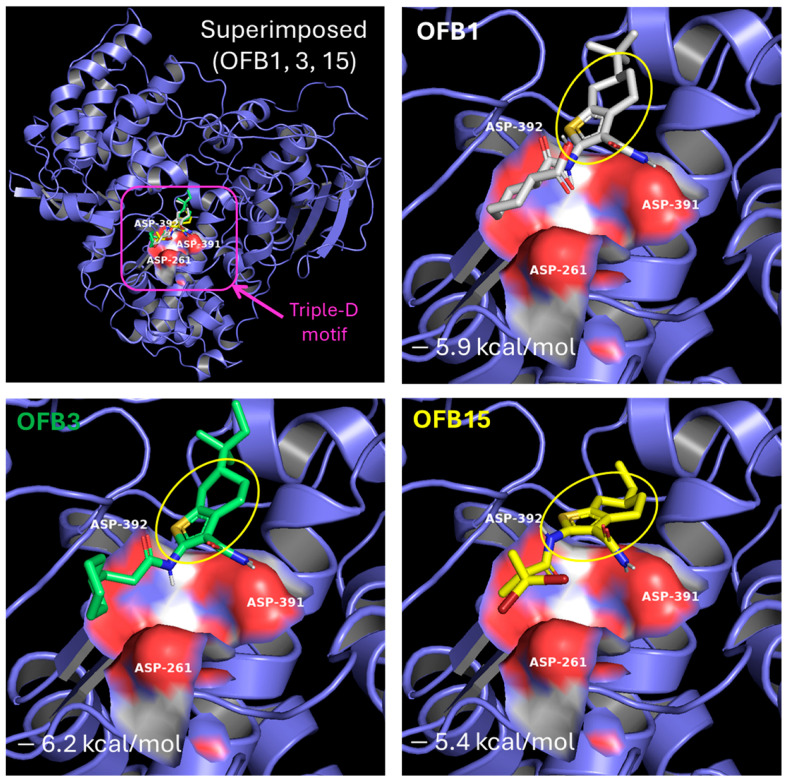
Molecular docking of OFB1, OFB3, and OFB15 to the Triple-D (Asp) motif of the POLcon active site using the AutoDock Vina program [22,23]. The amino acids of the Triple-D (Asp) motif, D535 (D261), D665 (D391), and D666 (D392) are shown by the red surface at the Active site (magenta square). The common Benzothiophene-based group structure is circled in yellow. The docking scores (kcal/mol) are also labeled for each compound.

Comparing the structures of these three inhibitors, they all have a common structure of Benzothiophene (Figure 7). The molecular docking of OFB1, OFB3, and OFB15 was conducted using the AutoDock Vina program, and the results are shown in Figure 8. According to molecular docking, all these three compounds could dock to the Triple-D motif in the Active site of RdRp. The Benzothiophene appears to be playing a major role in contact with the Triple-D motif. The differences at the position-6 group of Benzothiophene may have contributed to the activity differences among these compounds against different viruses. It is suggested that Benzothiophene derivates could bind to the Triple-D motif at the activate site of RdRp to block viral replication. To our knowledge, there are no reports of Benzothiophene derivatives for anti-flavivirus infection.

## 4. Discussion

Based on the virtual screening against the target of the Triple-D motif, the Benzothiophene-derived compounds OFB1, OFB3, and OFB15 are expected to target the Triple-D motif in the active site of RdRp to interfere with viral genome replication. Further molecular docking analysis demonstrated that these compounds have a high affinity for binding to the Triple-D motif and preventing viral RNA synthesis and replication. This finding will provide a new class of compounds for drug design and development against flavivirus infections.

According to the structures of these three compounds, we observed a common element of Benzothiophene. These S-bicyclic rings may play a vital role in interacting with the binding site in the RdRps. From molecular docking, it has been revealed that OFB compounds could interact with the negative charge surface of the Triple-D motif through electrostatic force, such as by forming hydrogen bonds. These electrostatic attractive interactions will be helpful for conducting the Benzothiophene-based design to achieve potent compounds against flaviviruses. For example, the extended methyl group, cyclohexane ring or both of compound OFB3 may have contributed to inhibiting WNV. More extensive screening against this Trible-D motif of POLcon will be conducted to find more potent common lead compounds for drug development against flaviviruses.

Benzothiophene is a natural aromatic organic compound with the molecular formula C_8_H_6_S and has a bioactive structure property used as a scaffold for chemical synthesis. Benzothiophene derivatives have been widely used as pharmaceutical drugs [29,30,31], but there are few reports for antivirals [32,33]. Interestingly, a similar class of pyridobenzothiazole derivatives was reported to have activities against HCV, DENV, and WNV, which also act by targeting the RdRp at the Active site [34,35].

The OFB compounds exhibited activities against different flaviviruses. All these three OFB compounds (OFB1, OFB3, and OFB15) showed strong inhibition of ZIKV, but weaker for DENV (DENV-2), and only OFB3 can neutralize WNV. Although we have not tested JEV and YFV, this report has provided a proof-of-concept that developing common or shared drugs is possible. The conserved common structure of POLcon has offered a substantial base for drug targeting, and especially the negatively charged Triple-D motif, which may have advantages for positively charged binders.

In conclusion, using a common RdRp structure of POLcon, we have identified a novel class of Benzothiophene derivates as inhibitors for flaviviruses. It has been demonstrated that POLcon is a promising target for finding shared drugs against different flaviviruses. Certainly, we will continue working on this target to identify more potent leading compounds for anti-flavivirus drug development.

## Data Availability

The original data presented in the study are included in the article/Supplementary Materials; further inquiries can be directed to the corresponding author.

## Supplementary Materials

The following supporting information can be downloaded at: https://www.mdpi.com/article/10.3390/v17020145/s1, Table S1: List of all compounds tested.

## References

[1] Postler T.S., Beer M., Blitvich B.J., Bukh J., de Lamballerie X., Drexler J.F., Imrie A., Kapoor A., Karganova G.G., Lemey P. (2023). Renaming of the genus Flavivirus to Orthoflavivirus and extension of binomial species names within the family Flaviviridae. Arch. Virol..

[2] Best S.M. (2016). Flaviviruses. Curr. Biol..

[3] Hastings A.K., Fikrig E. (2017). Zika Virus and Sexual Transmission: A New Route of Transmission for Mosquito-borne Flaviviruses Yale. J. Biol. Med..

[4] Diani E., Lagni A., Lotti V., Tonon E., Cecchetto R., Gibellini D. (2023). Vector-Transmitted Flaviviruses: An Antiviral Molecules Overview. Microorganisms.

[5] Shi Y., Gao G.F. (2017). Structural Biology of the Zika Virus. Trends Biochem. Sci..

[6] Barrows N.J., Campos R.K., Liao K.C., Prasanth K.R., Soto-Acosta R., Yeh S.C., Schott-Lerner G., Pompon J., Sessions O.M., Bradrick S.S. (2018). Biochemistry and Molecular Biology of Flaviviruses. Chem. Rev..

[7] Godoy A.S., Lima G.M., Oliveira K.I., Torres N.U., Maluf F.V., Guido R.V., Oliva G. (2017). Crystal structure of Zika virus NS5 RNA-dependent RNA polymerase. Nat. Commun..

[8] Choi K.H., Rossmann M.G. (2009). RNA-dependent RNA polymerases from Flaviviridae. Curr. Opin. Struct. Biol..

[9] Hasan S.S., Sevvana M., Kuhn R.J., Rossmann M.G. (2018). Structural biology of Zika virus and other flaviviruses. Nat. Struct. Mol. Biol..

[10] Knyazhanskaya E., Morais M.C., Choi K.H. (2021). Flavivirus enzymes and their inhibitors. Enzymes.

[11] Malet H., Masse N., Selisko B., Romette J.L., Alvarez K., Guillemot J.C., Tolou H., Yap T.L., Vasudevan S., Lescar J. (2008). The flavivirus polymerase as a target for drug discovery. Antivir. Res..

[12] Maddipati V.C., Mittal L., Mantipally M., Asthana S., Bhattacharyya S., Gundla R. (2020). A Review on the Progress and Prospects of Dengue Drug Discovery Targeting NS5 RNA-Dependent RNA Polymerase. Curr. Pharm. Des..

[13] Shimizu H., Saito A., Mikuni J., Nakayama E.E., Koyama H., Honma T., Shirouzu M., Sekine S.I., Shioda T. (2019). Discovery of a small molecule inhibitor targeting dengue virus NS5 RNA-dependent RNA polymerase. PLoS Negl. Trop. Dis..

[14] Garcia L.L., Padilla L., Castano J.C. (2017). Inhibitors compounds of the flavivirus replication process. Virol. J..

[15] Dubankova A., Boura E. (2019). Structure of the yellow fever NS5 protein reveals conserved drug targets shared among flaviviruses. Antivir. Res..

[16] Pattnaik A., Palermo N., Sahoo B.R., Yuan Z., Hu D., Annamalai A.S., Vu H.L.X., Correas I., Prathipati P.K., Destache C.J. (2018). Discovery of a non-nucleoside RNA polymerase inhibitor for blocking Zika virus replication through in silico screening. Antivir. Res..

[17] Bai F., Wang T., Pal U., Bao F., Gould L.H., Fikrig E. (2005). Use of RNA interference to prevent lethal murine west nile virus infection. J. Infect. Dis..

[18] Nazneen F., Thompson E.A., Blackwell C., Bai J.S., Huang F., Bai F. (2023). An effective live-attenuated Zika vaccine candidate with a modified 5′ untranslated region. npj Vaccines.

[19] Webb B., Sali A. (2016). Comparative Protein Structure Modeling Using MODELLER. Curr. Protoc. Protein Sci..

[20] Osawa T., Aoki M., Ehara H., Sekine S.I. (2023). Structures of dengue virus RNA replicase complexes. Mol. Cell.

[21] Biswal M., Yao W., Lu J., Chen J., Morrison J., Hai R., Song J. (2024). A conformational selection mechanism of flavivirus NS5 for species-specific STAT2 inhibition. Commun. Biol..

[22] Eberhardt J., Santos-Martins D., Tillack A.F., Forli S. (2021). AutoDock Vina 1.2.0: New Docking Methods, Expanded Force Field, and Python Bindings. J. Chem. Inf. Model..

[23] Trott O., Olson A.J. (2010). AutoDock Vina: Improving the speed and accuracy of docking with a new scoring function, efficient optimization, and multithreading. J. Comput. Chem..

[24] Schrödinger LLC (2015). The PyMOL Molecular Graphics System, Version 1.8. https://www.pymol.org/support.html.

[25] Duan W., Song H., Wang H., Chai Y., Su C., Qi J., Shi Y., Gao G.F. (2017). The crystal structure of Zika virus NS5 reveals conserved drug targets. EMBO J..

[26] Lim S.P., Noble C.G., Seh C.C., Soh T.S., El Sahili A., Chan G.K., Lescar J., Arora R., Benson T., Nilar S. (2016). Potent Allosteric Dengue Virus NS5 Polymerase Inhibitors: Mechanism of Action and Resistance Profiling. PLoS Pathog..

[27] Surana P., Satchidanandam V., Nair D.T. (2014). RNA-dependent RNA polymerase of Japanese encephalitis virus binds the initiator nucleotide GTP to form a mechanistically important pre-initiation state. Nucleic Acids Res..

[28] Malet H., Egloff M.P., Selisko B., Butcher R.E., Wright P.J., Roberts M., Gruez A., Sulzenbacher G., Vonrhein C., Bricogne G. (2007). Crystal structure of the RNA polymerase domain of the West Nile virus non-structural protein 5. J. Biol. Chem..

[29] Penthala N.R., Sonar V.N., Horn J., Leggas M., Yadlapalli J.S., Crooks P.A. (2013). Synthesis and evaluation of a series of benzothiophene acrylonitrile analogs as anticancer agents. Medchemcomm.

[30] Banerjee T., Kapoor N., Surolia N., Surolia A. (2011). Benzothiophene carboxamide derivatives as novel antimalarials. IUBMB Life.

[31] De Vreese R., Van Steen N., Verhaeghe T., Desmet T., Bougarne N., De Bosscher K., Benoy V., Haeck W., Van Den Bosch L., D’Hooghe M. (2015). Synthesis of benzothiophene-based hydroxamic acids as potent and selective HDAC6 inhibitors. Chem. Commun..

[32] Boulware S.L., Bronstein J.C., Nordby E.C., Weber P.C. (2001). Identification and characterization of a benzothiophene inhibitor of herpes simplex virus type 1 replication which acts at the immediate early stage of infection. Antivir. Res..

[33] Makino M., Azuma M., Wakamatsu S.I., Suruga Y., Izumo S., Yokoyama M.M., Baba M. (1999). Marked suppression of T cells by a benzothiophene derivative in patients with human T-lymphotropic virus type I-associated myelopathy/tropical spastic paraparesis. Clin. Diagn. Lab. Immunol..

[34] Tarantino D., Cannalire R., Mastrangelo E., Croci R., Querat G., Barreca M.L., Bolognesi M., Manfroni G., Cecchetti V., Milani M. (2016). Targeting flavivirus RNA dependent RNA polymerase through a pyridobenzothiazole inhibitor. Antivir. Res..

[35] Manfroni G., Meschini F., Barreca M.L., Leyssen P., Samuele A., Iraci N., Sabatini S., Massari S., Maga G., Neyts J. (2012). Pyridobenzothiazole derivatives as new chemotype targeting the HCV NS5B polymerase. Bioorg. Med. Chem..

