# Supplementary material for: Identification of Benzothiophene-Derived Inhibitors of Flaviviruses by Targeting RNA-Dependent RNA Polymerase
Source: Viruses. 2025 Jan 23;17(2):145. doi: 10.3390/v17020145 (PMC11861172; doi:10.3390/v17020145)
Supplement: Supplementary file 1 [file viruses-17-00145-s001.zip › viruses-3417277-supplementary.pdf]

Table S1 List of compounds tested

OFB#	ID	Structure	Mol Weight	Mol Name
1	6111542	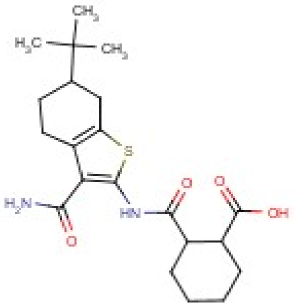	406.5	2-({[3-(aminocarbonyl)-6-tert-butyl-4,5,6,7-tetrahydro-1-benzothien-2-yl]amino}carbonyl)cyclohexanecarboxylic acid
2	6131364	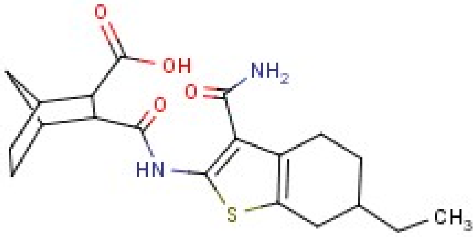	390.5	3-({[3-(aminocarbonyl)-6-ethyl-4,5,6,7-tetrahydro-1-benzothien-2-yl]amino}carbonyl)bicyclo[2.2.1]heptane-2-carboxylic acid
3	5695655	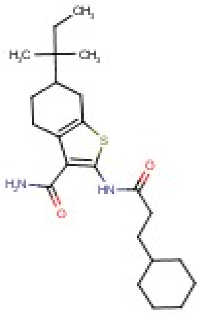	404.6	2-[(3-cyclohexylpropanoyl)amino]-6-(1,1-dimethylpropyl)-4,5,6,7-tetrahydro-1-benzothiophene-3-carboxamide
4	6129917	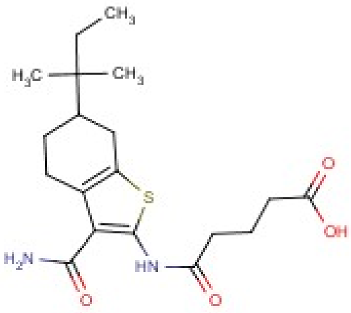	380.5	5-{[3-(aminocarbonyl)-6-(1,1-dimethylpropyl)-4,5,6,7-tetrahydro-1-benzothien-2-yl]amino}-5-oxopentanoic acid

5	5245275	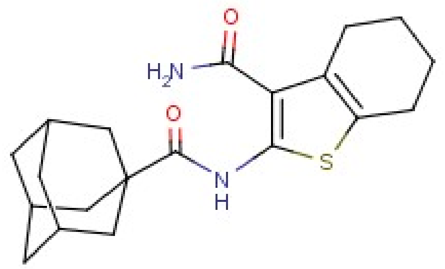	358.5	2-[(1-adamantylcarbonyl)amino]-4,5,6,7-tetrahydro-1-benzothiophene-3-carboxamide
6	8823264	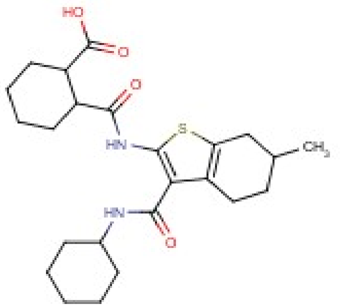	446.6	2-[({3-[(cyclohexylamino)carbonyl]-6-methyl-4,5,6,7-tetrahydro-1-benzothien-2-yl}amino)carbonyl]cyclohexanecarboxylic acid
7	7314826	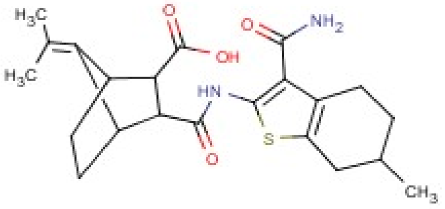	416.5	3-({[3-(aminocarbonyl)-6-methyl-4,5,6,7-tetrahydro-1-benzothien-2-yl]amino}carbonyl)-7-(1-methylethylidene)bicyclo[2.2.1]heptane-2-carboxylic acid
8	8821758	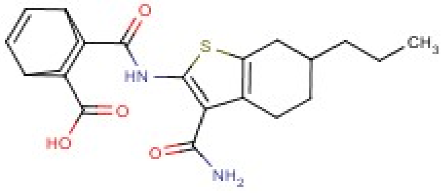	416.5	3-({[3-(aminocarbonyl)-6-propyl-4,5,6,7-tetrahydro-1-benzothien-2-yl]amino}carbonyl)bicyclo[2.2.2]oct-5-ene-2-carboxylic acid

9	8823352	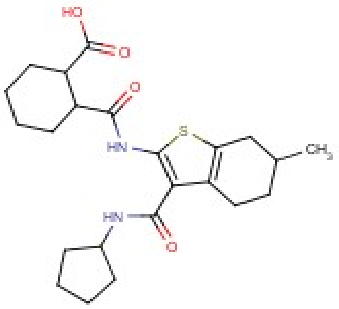	432.6	2-[({3-[(cyclopentylamino)carbonyl]-6-methyl-4,5,6,7-tetrahydro-1-benzothien-2-yl}amino)carbonyl]cyclohexanecarboxylic acid
10	8823254	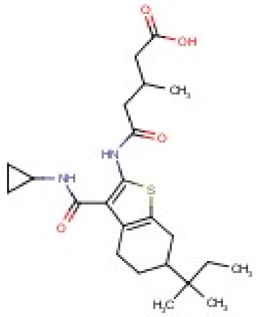	434.6	5-{[3-[(cyclopropylamino)carbonyl]-6-(1,1-dimethylpropyl)-4,5,6,7-tetrahydro-1-benzothien-2-yl]amino}-3-methyl-5-oxopentanoic acid
11	8824221	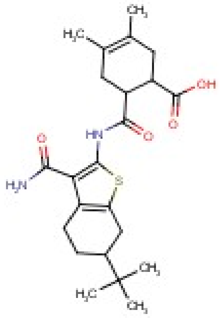	432.6	6-({[3-(aminocarbonyl)-6-tert-butyl-4,5,6,7-tetrahydro-1-benzothien-2-yl]amino}carbonyl)-3,4-dimethyl-3-cyclohexene-1-carboxylic acid
12	8823258	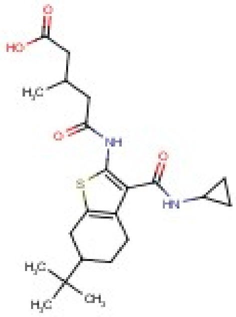	420.6	5-({6-tert-butyl-3-[(cyclopropylamino)carbonyl]-4,5,6,7-tetrahydro-1-benzothien-2-yl}amino)-3-methyl-5-oxopentanoic acid

13	8823410	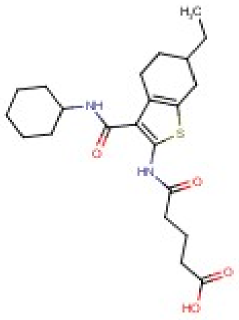	420.6	5-({3-[(cyclohexylamino)carbonyl]-6-ethyl-4,5,6,7-tetrahydro-1-benzothien-2-yl}amino)-5-oxopentanoic acid
14	8823463	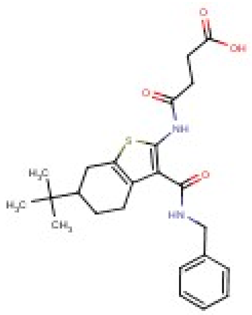	442.6	4-({3-[(benzylamino)carbonyl]-6-tert-butyl-4,5,6,7-tetrahydro-1-benzothien-2-yl}amino)-4-oxobutanoic acid
15	8814541	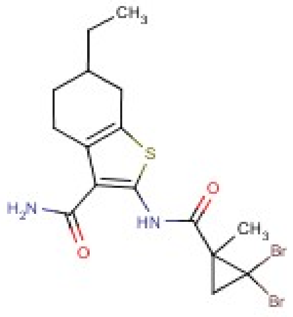	464.2	2-{[(2,2-dibromo-1-methylcyclopropyl)carbonyl]amino}-6-ethyl-4,5,6,7-tetrahydro-1-benzothiophene-3-carboxamide
16	7353927	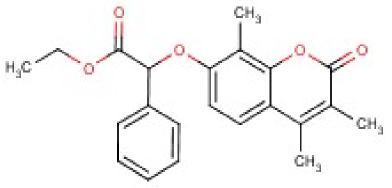	366.4	ethyl phenyl[(3,4,8-trimethyl-2-oxo-2H-chromen-7-yl)oxy]acetate

17	7660228	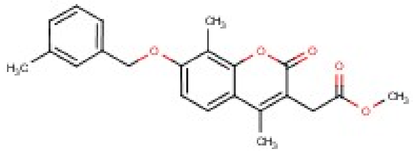	366.4	methyl {4,8-dimethyl-7-[(3-methylbenzyl)oxy]-2-oxo-2H-chromen-3-yl}acetate
